# Ru (III) Catalyzed Oxidation of Aliphatic Ketones by N-Bromosuccinimide in Aqueous Acetic Acid: A Kinetic Study

**DOI:** 10.1100/2012/456516

**Published:** 2012-04-30

**Authors:** P. Giridhar Reddy, K. Ramesh, S. Shylaja, K. C. Rajanna, S. Kandlikar

**Affiliations:** Department of Chemistry, Osmania University, Hyderabad 500 007, India

## Abstract

Kinetics of Ru (III) catalyzed oxidation of aliphatic ketones such as acetone, ethyl methyl ketone, diethyl ketone, iso-butylmethyl ketone by N-bromosuccinimide in the presence of Hg(II) acetate have been studied in aqueous acid medium. The order of [N-bromosuccinimide] was found to be zero both in catalyzed as well as uncatalyzed reactions. However, the order of [ketone] changed from unity to a fractional one in the presence of Ru (III). On the basis of kinetic features, the probable mechanisms are discussed and individual rate parameters evaluated.

## 1. Introduction

N-bromosuccinimide (NBS) has been used as a brominating and oxidizing agent in synthetic organic chemistry as well as analytical reagent especially in acid medium [1–25]. Recently, NBS has been used for the bromination of some selected organic substrates in which it is used as source for bromine in radical reactions (such as allylic brominations) and various electrophilic additions. The NBS reaction with organic substrates such as alcohols and amines leads to the products of net oxidation followed by elimination of HBr [7–17]. During the past few decades, there has been an upsurge in the designing of a variety of catalysts to explore their utility in synthetic organic chemistry. A number of transition and platinum group metal ions and their complexes have been designed and used as catalysts under homogeneous conditions. Microconcentration of Ru (III) has been found to be an efficient catalyst in a number of redox systems that are reported from our laboratories and elsewhere [26–32]. However, there seems to be no report on Ru (III)-mediated oxidation of ketones by NBS even though few reports are available under uncatalyzed conditions [33–35] by N-bromo compounds. In view of the above, we have studied the kinetics of oxidation of few aliphatic ketones by NBS in aqueous acetic acid solutions under catalytic conditions using microconcentrations of Ru (III) as catalyst.

## 2. Experimental

### 2.1. Reagents

All the chemicals used are of analytical grade. Acetic acid was refluxed with chromic oxide and acetic anhydride for 6 h and then fractionally distilled according to literature procedures [36].

### 2.2. Stoichiometry of the Reaction

Stoichiometry of the reaction was determined by taking known excess of NBS over [ketone] in aqueous acid media at a constant ionic strength (*μ*) and desired temperature. The progress of the reaction was followed for several days to ensure the completion of the reaction. The unreacted [NBS] in aliquots (5 mL each) was estimated every day till a constancy in the titer value is obtained. Final analysis indicated that *one mole* of Ketone consumed *two moles* of NBS.

### 2.3. Kinetic Method

All kinetic measurements were performed under pseudo-first-order conditions with [Ketone] at least 10-fold in excess over [NBS] at a constant ionic strength (*μ*) and desired temperature. The reaction was initiated by mixing previously thermostated solutions of NBS and Ketone, which also contained necessary quantities of acid and NaClO_4_. The progress of the reaction was followed by iodometric determination of the unreacted [NBS] in aliquots (5 mL each) of the reaction mixture withdrawn into aqueous KI solutions at regular time intervals. The initial rates (*V*) were evaluated from the tangential slopes of the plots of [NBS] versus time. The initial rate (*V*) values were reproducible within *±*5%. The oxidation product was identified as 1,2-dicarbonyl compound according to standard procedures cited in literature [37]. 

The effect of dissolved oxygen on the rate of the reaction was studied by preparing the reaction mixture and following the reaction under nitrogen atmosphere. No significant difference between the results obtained under nitrogen atmosphere and those obtained in the presence of atmospheric oxygen was observed. However, fresh solutions were used during the experiments.

## 3. Results and Discussion

### 3.1. Effect of Variation of [substrate]

**Figure 1:**
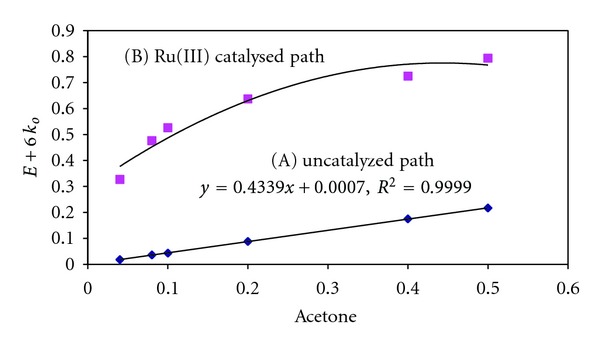
Plot of *k*_*o*_ versus [acetone] in NBS-acetone reaction 10^2^ [Hg(OAc)_2_] = 2.00 mol dm^−3^; HOAc = 10% (v/v); 10^2^ [H+] = 5.00 mol dm^−3^; 10^5^ [Ru (III)] = 2.09 mol dm^−3^; temp = 300 K.

**Figure 2:**
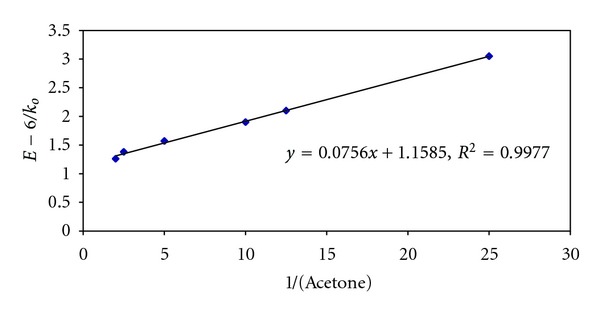
Plot of 1/*k*_*o*_ versus 1/[acetone] in Ru (III) catalyzed NBS-acetone reaction. 10^2^ [Hg(OAc)_2_] = 2.00 mol dm^−3^; HOAc = 10% (v/v); 10^2^ [H^+^] = 5.00 mol dm^−3^; 10^5^ [Ru (III)] = 2.09 mol dm^−3^; temp = 300 K.

Acetone (MMK), ethyl methyl ketone (EMK), and iso-butyl methyl ketone (IBMK) were used as substrates in the present study. The rate of oxidation increased with an increase in [Substrate] and a first-order dependence in [Substrate] was observed. However, in the presence of Ru (III), the order with respect to [S] changed from unity to a fraction (Table 1).  Figure 1 shows the plots of *k*_0_ versus [Acetone] for uncatalyzed and Ru (III) catalyzed reactions. The plot of uncatalyzed reaction is linear with an excellent correlationship (*R* ^2^ = 0.999) while the catalyzed plot is a nonlinear curve much above the uncatalyzed plot. However, the reciprocal plot of (1/*k*_0_) versus (1/[Ketone]) is linear with excellent correlationship (*R* ^2^ = 0.999) as shown in Figure 2. This observation indicates the formation of intermediate complex [Ru (III)-Substrate].

### 3.2. Effect of Variation of [Ru (III)]

Highly significant rate (*V*) accelerations were observed with an increase in [Ru (III)] and the order with respect to [Ru (III)] was found to be unity (Table 2).

### 3.3. Effect of Variation of Acidity

The reaction rates increased with an increase in acidity ([H^+^]) at constant ionic strength *μ* in uncatalysed system, that is, first-order dependence on [H^+^], whereas in the case of Ru (III)-catalyzed system, [H^+^] effect is negligible (Table 3).

### 3.4. Effect of Variation of Mercuric Acetate

A four-fold change (0.02 to 0.08 M) in the concentration of mercuric acetate did not affect the rate of reaction to any considerable extent.

### 3.5. Effect of Variation of Solvent Composition

The reaction rates were found to increase with an increase in the percentage of acetic acid (Table 4). The increase in the rate of oxidation with increase in the polarity of the medium suggests a more polar transition state than the reactants. The plot of log⁡*k* versus the inverse of the relative permittivity is nonlinear. The solvent effect was also analyzed using Grunwald-Winstein equation as cited in literature [38, 39]:

(1) $$\log k=\log k_{0}+mY.$$

The plot of log⁡*k* versus *Y* is linear (*r* = 0.9994) with *m* = 0.77 ± 0.02. The value of *m* suggests a more polar transition state than the reactants. Thus, a considerable charge separation takes place in the transition state of the reaction.

### 3.6. Effect of Variation of Temperature

Effect of variation of temperature has been studied to compute activation and thermodynamic parameters. The enthalpy of activation (Δ*H* ^#^) and entropy of activation (Δ*S* ^#^) are computed from Eyring plots of ln⁡(*k*/*T*) versus (1/*T*) (Figures 4 and 5) while free energy of activation (Δ*G* ^#^) is obtained from Gibbs-Helmholtz relationship. The data of activation parameters have been compiled in Tables 5 and 6. The enthalpy (Δ*H*) and entropy (Δ*S*) of [Ru (III)-S] complex formation have been obtained from vant Hoff's equation, while free energy of formation is obtained from vant Hoff's reaction isotherm.

### 3.7. Mechanism of Oxidation in Uncatalyzed Reaction

In order to gain an insight into the mechanistic path, it is essential to know the nature of the reactive oxidizing and reducing species. NBS is known to exist in three forms in acid media, namely, NBS itself, NBSH^+^ (protonated NBS) and bromonium ion (Br^+^). Since all the kinetic studies are conducted in the presence of mercuric acetate, the liberated Br_2_ during the course of the reaction according to (2) can be removed in the form of HgBr_4_^2−^ or HgBr_2_ complexes because Hg (OAc)_ 2_ acts as a scavenger for Br^−^ formed in the reaction:

(2) $$\begin{array}{c} >\text{NBr}+\text{HBr}\to >\text{NH}\;+\;{\text{Br}}_{2}\ldots . \end{array}$$

In acid media, ketones are protonated to yield oxonium salts. Since oxygen is more electronegative than carbon, the first resonating structure (I) makes a larger contribution than the second (II):

(3) $$\begin{array}{cccc} & >\text{C}=\text{O}+{\text{H}}^{+} & ⟷>\text{C}={\text{O}}^{+}–\text{H} & ⟷>{\text{C}}^{+}–\text{O}–\text{H}\ldots .\\ & & \text{I} & \text{II} \end{array}\;\;$$

**Scheme 1:**
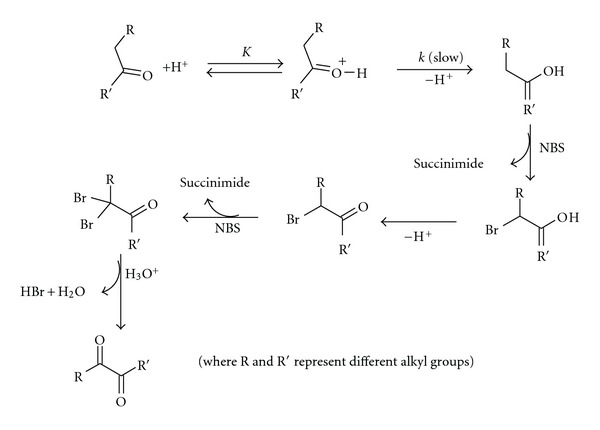


This discussion together with the observed kinetics, namely, first-order dependence on [ketone] as well as [H^+^] and zero-order dependence on [NBS] substantiate a mechanism comprising the enolisation step prior to the slowest step as shown in Scheme 1. 

This mechanism is similar to the one proposed by Litter and Waters [14–17] in the case of two electrons abstracting oxidation process. Participation of ion in the rate-limiting step can be further supported from negligible salt effect and enhanced rates in the solvents of low dielectric constant. Rate law, for the above mechanism, comes out as

(4) $$-d\frac{\left[\text{NBS}\right]}{dt}=k^{\prime \prime }\;\left[\text{ketone}\right]\left[{\text{H}}^{+}\right]\ldots ,$$

where *k*′′ = *kK*. This rate expression is consistent with observed kinetics, namely, zero order with respect to [NBS] and first order in [ketone] as well as [H^+^]. The kinetic and activation parameters presented in the table indicate that the second-order rate constant *k*′′ is almost doubled with a 10° (*ten degrees*) rise in temperature. The free energy of activation Δ*G* ^#^ of the present study fits well with the one reported by [18–25, 33] (23.0 K cal/mol) in the oxidation of diethyl ketone by NBS in acid medium which depicts the validity of a similar mechanism being operative in the NBS oxidation of ethyl methyl ketone (EMK) and iso-butyl methyl ketone (IBMK). A perusal of kinetic constants compiled in Table 5 indicates that the rate of oxidation follows the sequence: IBMK > EMK > MMK which may be attributed to the increased +I effect (inductive effect) due to an increase in electron cloud in the vicinity of alkyl groups present in EMK and IBMK. This can be also evidenced by Δ*S* ^#^ values (Table 5) which follow the order: IBMK > EMK > MMK. By and large Δ*H* ^#^ values also show a similar trend. Then, the trend exhibited by Δ*S* ^#^ agrees with that of second-order rate constants. Thus it is not unjustifiable to say that the reactions are entropy controlled. 

### 3.8. Mechanism of Oxidation in the Presence of Ru (III)

The absence of free radicals, zero-order dependence on [oxidant] and that in [ketone] is changed from unity to a fraction from the uncatalysed system to Ru (III)-catalyzed system [18–25]. Such a change in the reaction order of [S] from uncatalyzed to catalyzed system usually points out the intermediate complex formation either with metal ion or NBS itself, but the zero-order dependence of NBS rules out complexation between NBS and ketone. Ultimately, one can visualize the complex formation of ketone with Ru (III). The Ru (III) employed in the system are known to form adduct with organic compounds due to the partially filled d-orbitals. Complexation between ketone and Ru (III) appears, therefore, most likely. Negligible acidity and salt effects probably suggest the keto form to be active in the study. However, the solvent effect studies point out that the reaction rate is faster in the solvent of low dielectric constant thus indicating the participation of ionic species in the rate limiting step. The most probable mechanism in Ru (III)-catalyzed system can be traced as in Scheme 1 by taking ethyl methyl ketone as a typical substrate.

**Scheme 1**

(5) $$\begin{array}{c} \text{EMK}+\text{Ru}\left(\text{III}\right)\overset{k}{↔}\left[\text{EMK-Ru}\left(\text{III}\right)\right],\\ \left[\text{EMK-Ru}\left(\text{III}\right)\right]\underset{\text{slow}}{\overset{\;\;k\;\;}{\to }}\text{Ru}\left(\text{I}\right)+P_{1},\\ P_{1}+\text{NBS}\overset{\text{fast}}{\to }\text{Products}+\text{HBr}+\text{NHS},\\ \text{Ru}\left(\text{I}\right)+\text{NBS}\overset{\text{fast}}{\to }\text{NHS}+\text{Ru}\left(\text{III}\right)+\text{HBr}, \end{array}$$

where *P*_1_: Carbonium ion intermediate. Rate law for the mechanism was derived as

(6) $$\begin{array}{c} -d\frac{\left[\text{NBS}\right]}{dt}=\frac{kK\left[\text{Ru}\left(\text{III}\right)\right]\left[\text{EMK}\right]}{1+K\left[\text{EMK}\right]}\ldots . \end{array}$$

**Figure 3:**
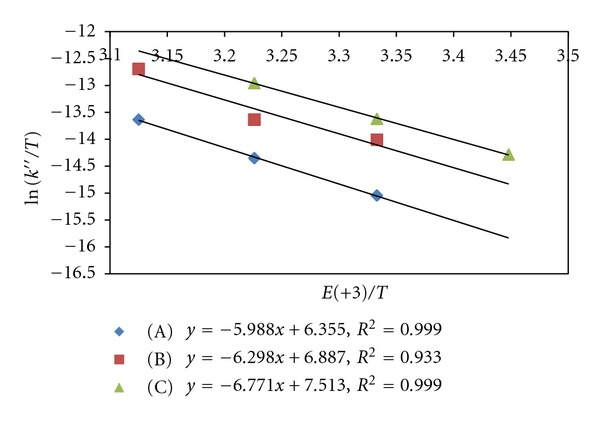
Eyring's plot of ln⁡(*k*′′/*T*) versus 1/*T* (uncatalyzed reaction). (A) Acetone (B) EMK (C) IBMK.

**Figure 4:**
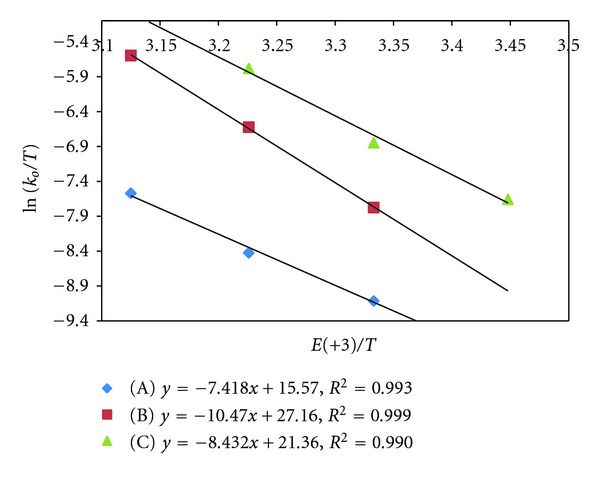
Eyring's plot of ln⁡(*k*_*o*_/*T*) versus 1/*T* for Ru (III) catalyzed reaction. Reaction (A) Acetone (B) EMK (C) IBMK.

**Figure 5:**
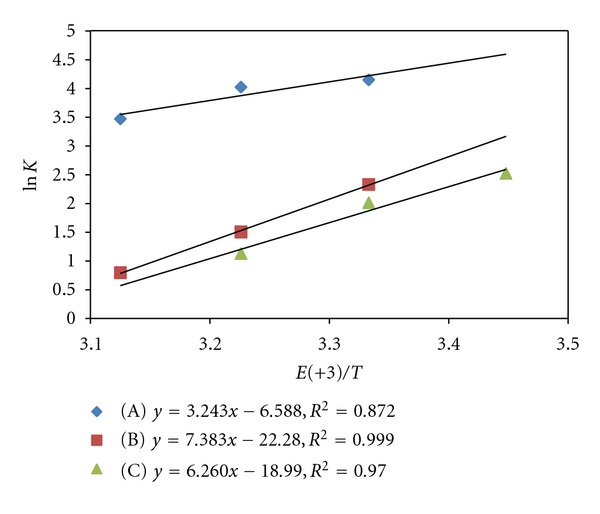
vant Hoff's plot of ln⁡*K* versus 1/*T* for Ru (III) catalyzed reaction. (A) Acetone (B) EMK (C) IBMK.

The reciprocals of (6) demand that plot of (1/*k*_0_) versus (1/[Ketone]) should be linear with positive slope and intercept on ordinate. Such plots have been realized in this study with excellent correlationship (*R* ^2^ = 0.999) as shown in Figure 2, showing the validity proposed mechanism given in Scheme 1. The decomposition constant (*k*) and formation constant (*K*) for [Ru (III)-Ketone] adducts are evaluated from the intercept and slopes of these plots. Thermodynamic parameters pertaining to the formation constant are evaluated from known procedures and compiled in Table 6. It is interesting to note that Ru (III)-catalyzed rate of oxidation of IBMK is almost ten times greater than that of acetone (MMK) as evidenced from the magnitude of decomposition constant (*k*) values (IBMK > EMK > MMK presented in Table 6). This may be explained due to the increased + I effect due to an increased electron cloud of alkyl group in IBMK compared to MMK or EMK. However, this trend cannot be supported by either enthalpy of activation (Δ*H* ^#^) or entropy of activation (Δ*S* ^#^) because none of these parameters indicated a regular pattern, but according to Hinshelwood's classification of reaction series, it appears that both Δ*H* ^#^ and Δ*S* ^#^ is important in controlling the reaction rate in the present study. Further, the negative entropies if activation (Δ*S* ^#^) data presented in Table 5 show that the transition state of uncatalyzed reaction is by large rigid due to salvation (Figure 3).

## 4. Conclusions

In conclusion, rate of oxidation of aliphatic ketones such as acetone, ethyl methyl ketone, iso-butylmethyl ketone by N-bromosuccinimide (NBS) has been dramatically accelerated by trace amounts (micro concentrations) of Ru (III) in acid medium. Kinetic studies indicated zero order in [NBS] in Ru (III)-catalyzed as well as uncatalyzed reactions. However, the order of [Ketone] changed from unity to a fraction in the presence of Ru (III). Mechanism of oxidation was explained through the formation of [Ru (III)-Ketone] precursor prior to rate-limiting step. According to Hinshelwood's classification of reaction series, present set of reactions appear to fall into the third category of reaction series, in which both Δ*H* ^#^ and Δ*S* ^#^ are important in controlling the reaction rate.

## Figures and Tables

**Table 1:** Effect of variation of [substrate] 10^2^ [Hg(OAc)_2_] = 2.00 mol dm^−3^; HOAc = 10% (v/v); 10^2^[*H* ^+^] = 5.00 mol dm^−3^; 10^5^ [Ru (III)] = 2.09 mol dm^−3^; temp = 300 K.

10^3^[NBS]	[ketone]	10^6^ *k*_*o*_ mol dm^−3^ s^−1^			
mol dm^−3^		EMK		IBMK	
2.00	0.040	0.050	(0.761)	0.725	
4.00	0.040	0.051	(0.759)	0.723	
5.00	0.040	0.049	(0.762)	0.728	
8.00	0.040	0.050	(0.760)	0.720	
5.00	0.080	0.102	(1.190)	1.40	
5.00	0.100	0.124	(1.330)	1.82	(3.03)
5.00	0.200	0.252	(1.790)	3.62	(4.17)
5.00	0.400	0.503	(2.130)	7.20	(5.00)
5.00	0.500			9.09	(5.55)

*Values presented in the parenthesis indicate Ru (III)-catalyzed redox system.

**Table 2:** Effect of variation of [Ru (III)]. 10^3^ [NBS] = 5.00 mol dm^−3^; 10^2^ [H^+^] = 1.00 mol dm^−3^; [EMK] = 0.100 mol dm^−3^; 10^2^ [Hg(OAc)_2_] = 2.00 mol dm^−3^; HOAc = 10% (v/v); temp = 300 K.

10^5^ [Ru (III)]	0.525	1.05	2.09	4.18	5.25
10^6^ V mol dm^−3^ s^−1^	0.338	0.675	1.35	2.72	3.40
10^6^ V/10^5^ [Ru (III)]	0.644	0.595	0.646	0.650	0.648

**Table 3:** Effect of variation of [H^+^]. 10^3^ [NBS] = 5.00 mol dm^−3;^ 10^2^ [Hg(OAc)_2_] = 2.00 mol dm^−3^; HOAc = 10% (v/v); [EMK] = 0.100 mol dm^−3^; Temp = 300 K; 10^5^ [Ru (III)] = 2.09 mol dm^−3^.

[H^+^] mol dm^−3^	0.010	0.025	0.050	0.075	0.100
10^7^ V mol dm^−3^ s^−1^	0.250	0.520	0.124	1.75	2.50
	(13.3)	(13.0)	(13.3)	(13.5)	(13.0)

*Values presented in the parenthesis indicate Ru (III)-catalyzed system.

**Table 4:** Effect of variation of solvent composition. 10^3^ [NBS] = 5.00 mol dm^−3^; [EMK] = 0.100 mol dm^−3^; Temp = 300 K 10^2^ [Hg(OAc)_2_] = 2.00 mol dm^−3^; 10^6^ [Ru (III)] = 5.25 mol dm^−3^.

HOAc % (v/v)	10	20	30	40	50
10^6^ *k*_0_ mol dm^−3^ s^−1^	0.124	0.175	0.220	0.270	0.325
	(0.340)	(0.385)	(0.435)	(0.480)	(0.535)

* Values presented in the parenthesis indicate Ru (III)-catalyzed system.

**Table 5:** Effect of temperature. Activation Parameters for unanalyzed system.

Parameter	T(K)	Acetone	Ethyl methyl ketone (EMK)	Isobutyl methyl ketone (IBMK)
10^4^ *k*′′(dm^3^ mol^−1^ s^−1^)	290	—	—	1.82
	300	0.088	0.248	3.66
	310	0.182	0.488	7.36
	320	0.384	0.986	—
Δ*H* ^#^ k J mol^−1^		56.4	54.9	135
Δ*G* ^#^ k J mol^−1^		102	102	93.1
Δ*S* ^#^ J K^−1^ mol^−1^		152	−152	140

**Table 6:** Kinetic and thermodynamic parameters involving rate constants and formation constants for Ru (III) catalyzed system.

Parameter	T(K)	Acetone	EMK	IBMK
*k* (dm^3^ mol^−1^ s^−1^)	290	—	—	0.137
	300	0.033	0.126	0.319
	310	0.068	0.412	0.957
	320	0.165	1.19	—
Δ*H* ^#^ k J mol^−1^		61.8	86.3	70.2
Δ*G* ^#^ k J mol^−1^		81.9	78.4	76.3
Δ*S* ^#^ J K^−1^ mol^−1^		−67.0	25.0	−20.3
*K* dm^3^ mol^−1^	290	—	—	12.5
	300	63.5	10.3	7.50
	310	56.0	4.51	3.10
	320	32.2	2.22	—
−Δ*H* k J mol^−1^		27.1	59.4	52.2
−Δ*G* k J mol^−1^		10.4	3.89	5.05
−Δ*S* J K^−1^ mol^−1^		55.6	179	157
